# Utility of Feed Enzymes and Yeast Derivatives in Ameliorating Deleterious Effects of Coccidiosis on Intestinal Health and Function in Broiler Chickens

**DOI:** 10.3389/fvets.2019.00473

**Published:** 2019-12-20

**Authors:** Elijah G. Kiarie, Haley Leung, Reza Akbari Moghaddam Kakhki, Rob Patterson, John R. Barta

**Affiliations:** ^1^Department of Animal Biosciences, University of Guelph, Guelph, ON, Canada; ^2^Department of Technical Services and Innovation, Canadian Bio-Systems Inc., Calgary, AL, Canada; ^3^Department of Pathobiology, University of Guelph, Guelph, ON, Canada

**Keywords:** *Eimeria*, coccidiosis, feed enzymes, intestinal health and function, feed efficiency, yeast derivatives

## Abstract

Coccidiosis induced necrotic lesions impair digestive capacity and barrier function in concurrence with increased risks for secondary bacterial infections. The industry has been successful in controlling coccidiosis with anticoccidials and vaccination. However, concerns over *Eimeria* species resistant to anticoccidials, gaps in vaccination and restriction on antibiotics is stimulating research and application of alternative and/or complimentary strategies for coccidiosis control. The aim of this paper is to appraise literature on the utility of feed enzymes and yeast derivatives in modulating coccidiosis. Feed enzymes can complement endogenous enzymes (protease, amylase, and lipase) that may become insufficient in coccidiosis afflicted birds. Coccidiosis in the upper small intestine creates conditions that enhances efficacy of phytase and there are reports indicating supplemental phytase can mitigate the negative impact of coccidiosis on bone quality. Increase in intestinal short chain fatty acids due supplemental fiber degrading enzymes has been linked with reduced survivability of *Eimeria*. There is evidence whole yeast (live or dead) and derivatives can modulate coccidiosis. Immunomudulation properties of the yeast derivatives have been shown to enhance cellular and humoral immunity in *Eimeria* challenge models which is critical for effectiveness of coccidial vaccination. Moreover, yeast nucleotides have been shown to be beneficial in stimulating healing of intestinal mucosal surface. Other novel work has shown that certain yeast cells can produce derivatives with anticoccidial compounds effective in attenuating oocysts shedding. Yeast cell surface has also been shown to be an effective oral *Eimeria* vaccine delivery vehicle. Overall, while further refinement research is warranted to address inconsistencies in responses and commercial application, there is evidence feed enzymes and yeast derivatives could complement strategies for maintaining intestinal function to bolster growth performance in broilers compromised with coccidiosis. However, broilers receive diets containing several feed additives with distinct mode of actions and yet there is dearth of empirical data on the expected responses.Future evaluations should consider combinations of additives to document animal responses and potential synergies.

## Introduction

Global human population is estimated to reach 9.6 billion by 2050, during this period, broiler chicken production is expected to grow by 121% to satisfy animal protein demand (1). However, animal protein production sector is under pressure to produce food products in ways that are ethical, environmentally sustainable, and wholesome. For example, animal agriculture uses significant amounts of antibiotics for therapy, prevention of bacterial infection, and growth promotion. There are growing concerns around the world on indiscriminate use of antibiotics and linkage to the emergence of antibiotic resistant pathogens. These concerns have necessitated consumer and legislative cessation and/or restrictions on use antibiotics for growth promotion (AGP). Moreover, there is a growing consumer demand for speciality poultry products reared on organic, all vegetable diet and pasture feeding regimens. These changes in the ways of poultry production are bringing new challenges and exacerbating old ones related to bird health, animal welfare, and regulations. In the context of poultry health and nutrition, the primary concerns are increased incidences of enteric diseases such as coccidiosis, necrotic enteritis, impaired nutrient digestion, and absorption ultimately leading to poor feed conversion efficiency as well as increased mortalities and condemnation at the processing plant. All these aspects converge to vindicate the importance of effective control and prevention of enteric pathogens to guarantee food safety and security for a growing human population.

Caused by protozoan parasites of the phylum apicomplexan, genus *Eimeria*, coccidiosis is inexplicably linked to the advancement and modernization of poultry production and annual global impact amounts to more than $3 billion in morbidity and mortality losses (2, 3). The protozoa invades intestinal cells as part of life cycle leading to impaired digestion and absorption, barrier function and secondary bacterial infections (4–6). The parasites exhibits remarkable species-specific sites of development and foci of pathology within the intestinal tract (5, 7–9). *Eimeria acervulina, E*. *maxima*, and *E*. *tenella* are the most frequently found species in commercial broiler chickens production systems (8). High animal densities seen in these production systems are favorable for transmission of *Eimeria* (8). *Eimeria* infection also exacerbates intestinal proliferation of pathogens such as *Clostridium, perfringens*, and *Salmonella enterica* serovars Enteritidis or *Typhimurium* (9–12). It follows that coccidiosis not only has implication on birds health but can also compromises food safety (13).

In recognition of the negative effects of coccidiosis in poultry production, the industry has long developed and adopted anticoccidials or live vaccination or combinations of these strategies for control (6, 14). However, concerns over *Eimeria* species resistant to anticoccidials and public concern over drug use in animal production is limiting chemotherapy options (15). Vaccination is dependent on optimal *Eimeria* cycling through each flock, is management intensive, and cross-protection to wild-type strains is not 100% effective (11, 16, 17). Moreover, vaccination involves provision of live *Eimeria* species within the first day of chick life which may increase the risk of enteric disturbances (18). There are numerous alternative feed additives to traditional coccidiosis control strategies that are claimed to attenuate, or remedy structural and functional intestinal damage occasioned by coccidiosis (19). The intent of this review is to appraise the body of published data on the role of feed enzymes and yeast derivatives in modulating coccidiosis.

## Feed Enzymes

The proposal of application of exogenous enzymes in poultry nutrition was initially suggested almost 100 years ago (20), however, the prohibitive cost did not allow their application in animal nutrition until many decades later (21). Xylanases and β-glucanases were pioneer commercial feed enzymes to deal with problematic viscous feedstuffs such as barley and wheat (22–25). Early experimentations showed that supplementation of these enzymes in diets rich in viscous feedstuffs improved digestibility, growth performance and reduced feed costs (21–23). These studies helped scientists to understand the modes of action and stimulated further research and development efforts to innovate novel activities targeting specific substrates and stabilized to withstand the rigors of feed processing and gastrointestinal conditions (26). Indeed, the utility of feed enzymes in non-ruminant nutrition is widely accepted (21–23, 25, 27). Feed enzymes are largely applied in monogastric feeding programs on the premise that animals are not able to digest 100% of dietary components. For example, broilers excrete 25–30% of ingested dry matter in the manure (28). This is because of anti-nutritional factors (ANF) such as phytic acid or indigestible fractions by the conditions and the array of digestive enzymes in the GIT (29, 30). Most commercial feed enzymes are developed and applied to target such ANF (25). Moreover, application in young birds is driven by the fact that the gastrointestinal tract is not well-developed because of (1) an immature immune system, (2) limited endogenous enzyme secretory capacity, and (3) unstable gut microbiota (31–33). Thus, the original uptake of feed enzyme technology in poultry nutrition was to degrade ANF in feedstuffs and to complement endogenous enzymes in gut of compromised animals particularly the newly hatched chicks.

## Evolving Role of Feed Enzymes in Poultry Nutrition

Pressure on feed costs is and will remain a decisive factor for profitable and sustainable poultry production, and feed enzymes have an established role in reducing feed costs by increasing the flexibility of feed ingredient choices. Moreover, the need to reduce nutrients excretion in animal protein value chain elevates the utility of feed enzyme in poultry operations. However, emerging issues such as the restriction on the use of AGP have stimulated new directions and perspectives for application of feed enzymes. Emerging evidence revolve around evaluation of feed enzymes as part of an integrated program of gut health management (24). The peculiarity is that intestinal microbiota nourishes on luminal nutrients (dietary and/or endogenous) (34). Due to differences in substrate preference and growth requirements, the composition and structure of the digesta largely influences GIT microbiome (24). It follows that, microbiome profile and metabolic function is partly reflective of feed composition (34). It is therefore plausible that manipulating diet digestibility will influence GIT microbiome (24). Furthermore, fiber degrading enzymes could release hydrolysis products “prebiotic” that can modulate intestinal microbiota (24, 35–37).

## Whole Yeast and Derivatives

Yeasts are unicellular, 5–10 μm in size, eukaryotic microorganisms belonging to fungi kingdom (38). Yeasts are important in many complex ecosystems and are involved in symbiotic, mutualistic, parasitic, and competitive interactions with other microorganisms. Since first observation by A. van Leeuwenhoek in 1680 and discovery of their function in fermentation by Louis Pasteur in 1850s, humans have exploited yeast for food and beverage production among many other applications for eons (39). Interestingly, although, there are more than 1,000 known species of yeast, very few are commercially exploited (40). The majority of yeast species are neither harmful nor beneficial, and few are known to be pathogenic to humans and/or animals (40). The genus *Saccharomyces* has ~20 species that are of significant industrial importance e.g., ethanol, bread, single cell protein, and vitamin production (40). The annual global production of *Saccharomyces cerevisiae* has been estimated to exceeds production of all other industrial microorganisms (41). *Candida utilis* (formerly classified as *Torulopsis utilis*) and commercially known as “Torula Yeast” is unique as it utilizes pentose sugars, making it very useful in processing wood pulp to paper. Another important yeast is *Kluyveromyces marxianus* or the “whey yeast” for dairy processing. Although commercial exploitation of yeast is largely on traditional fermentation processes, advancement in molecular biology has opened tremendous opportunities for developing yeast strains for diverse applications. For example, *Komagataella (Pichia) pastoris, S. cerevisiae, Ogataea (Hansenula) polymorpha*, for the heterologous production of proteins (40–42).

There are many yeast associated feed ingredients and feed additives that are produced, marketed, and applied in animal agriculture around the world (43). Major feed ingredients such as distillers' grains with solubles (DDGS), brewers yeast, whey yeast, and bakery co-products are derived from yeast fermentation processes (44, 45). Yeasts are used as rich sources of protein, minerals, vitamins (particularly B vitamins), and other nutrients for humans and animals. Production of single cell protein from yeast has been suggested to have tremendous advantages relative to plant, animal, and other microbial sources of protein because of their rapid growth rate on a wide variety of substrates, including industrial and agricultural waste (46). Moreover, the relatively large cell size and flocculation abilities of yeasts makes them easier to harvest than bacteria in fermentation media (46). Other speciality yeast products include yeast selenium and *Phaffia rhodozyma* yeast that improves flesh color in salmon and trout (43). Nutritional yeasts and products are used in feed industry as sources of amino acids and micronutrients (43). However, the utility of yeast products in animal agriculture has evolved to exploit their functional attributes. Of particular interest are the functional components of cell contents such as peptides, enzymes, nucleotides and cell wall constituents such as β-glucans, glycoproteins, mannans, and chitin (47, 48). Subsequent sections will briefly describe the functional attributes of yeast and derivatives that have been shown to influence health and immune status in poultry.

### Live Whole Yeast

Many yeast species are recognized safe by many regulatory authorities such as Qualified Presumption of Safety status assigned by the European Food Safety Authority, the Association of American Feed Control Officials and Canadian Food Inspection Agency. However, in general, most commercial probiotic feed additives for poultry are of bacterial preparations e.g., (49–53). The few non-bacterial (yeast or fungal) probiotics includes *Aspergillus oryzae* (54, 55), *Candida pintolopesii* (54), *Candida saitoana, Saccharomyces bourlardii* (56), and *S. cerevisiae* (57). Arguably, yeast-based probiotics are indispensable in ruminant nutrition for their effectiveness in modulating rumen microbiome (58, 59). Active dry yeast is one of the most common viable yeast used as a probiotic in livestock production. Yeast probiotics that are used in animal agriculture as feed additive products typically contain carrier materials such as limestone, rice hulls and/or distillers solubles. These products typically contain 5 × 10^9^ colony forming units per gram representing 20–25% of the CFU's of pure, active dry yeast (43). Commercial yeast-based probiotics are primarily manufactured in dry form and concerns have been raised over their stability in feed manufacturing processes (55, 60–62). For example, feed supplemented with active yeast cells was subjected to pelleting (82°C) or extrusion (72°C for 31s) (61). Pelleting did not affect total yeast counts but viable yeast numbers were reduced 10-fold. However, extrusion reduced both total and viable yeast counts. Majority of poultry diets are subjected to rigorous feed processing including particle size reduction and hydrothermal processing to improve feed efficiency and hygiene (26). Suggesting that survival of unprotected yeast cells would be expected to be low in poultry feed subjected to hydrothermal processing.

### Yeast Derivatives

Yeast derivatives are collectively referred to as yeast cultures and are largely composed of a combination of yeast biomass and fermentation products produced in conditioned fermentation processes. Traces of viable residual yeast cells may also be present. Their production entails inoculation of specific culture media with live yeast cells and subsequent fermentation under specific conditions, upon which the entire fermented media is subsequently dried. The harvested mass is often formulated into feed additive or subjected to downstream processing to produce speciality products. Production of speciality products is seen as a key differentiator of many yeasts based functional feed additives available to the poultry industry. As heterotrophic organisms, energy and carbon metabolism are intimately interconnected giving yeast cells ability to produce wide variety of derivatives depending on the composition of the fermentation media and the fermentation conditions (63). It follows that yeast culture production can be manipulated to produce unique feed additives that contain single or combination of derivatives beneficial to animal nutrition and health.

#### Enzymes

Enzymes were first discovered by French chemist Anselme Payen in 1833 (64). Decades later, Louis Pasteur concluded that fermentation was correlated with the life and organization of the yeast cells but not with the cell death (65). It can then be argued that yeasts were the pioneer organisms for enzymes production, however, the feed market is dominated by bacteria and filamentous fungi derived enzymes (21, 24, 25, 37). This is mainly because non-yeast microbial expression system are advantageous in terms of certain product and process developments (66). However, with advancement in biotechnology some yeasts for example, *K*. (Pichia) *pastoris, S. cerevisiae, O*. (Hansenula) *polymorpha*, and certain other yeast species have been developed for industrial production of enzymes and proteins (66–68). These refined yeast proteins are however, applied in the production of specialized chemicals such as pharmaceutical intermediates (69).

#### Nucleotides

The total nucleic acids concentration in whole yeast ranges from 3 to 12% dry cell weight (70, 71). Yeast are also rich in endogenous nucleases and proteases that can degrade nucleic acids, DNA, and RNA into nucleotides through autolysis (71, 72). By controlling pH, temperature, and duration as well as use of additives such as salt and exogenous enzymes, the yeast cell autolysis can be an optimized and standardized for consistent product quality (73). These modifications are increasingly being used to produce yeast nucleotide products for various industrial application. For example, under normal autolysis conditions, the RNA is mainly degraded to three primary nucleotides, however, under controlled enzymatic hydrolysis, 5 prime nucleotides of guanine, adenine, cytosine and uracil are produced (73, 74).

#### Cell Wall (YCW) Components

The YCW represents about 15 to 20% of yeast dry weight and are rich in β-glucans and mannans as well as traces of chitin. Structurally, YCW is made up of inner layer of insoluble β-glucans and mannans, middle layer of soluble β-glucans, and the external layer of glycoprotein (75). However, it should be noted these layers are not discrete but rather form complex structures that are recalcitrant to breakdown (75). β-1,3 glucans with β-1,6 branch linkages are the primary polysaccharide component in YCW and have been shown to display immune-modulating effects (76). In this context, there is increasing interest in refined extraction of β-glucans through mechanical (e.g., bead milling, sonication, high-pressure homogenization) and non-mechanical (e.g., thermolysis, osmotic shock, chemical, and enzymatic) methods (75, 77, 78). Mannans consist of α-1,6 bonds with side chains of mannose in α-1,2 bonds (79).

## Utility of Feed Enzymes and Yeast Derivatives in Mitigating Negative Effects of Coccidiosis

### Experimental Challenge Models

Production losses, increased mortality, reduced animal welfare and increased risk of contamination of poultry products due to enteric diseases is of great concern to the poultry industry (9, 10, 12, 13). There are many research investigations that used enteric pathogen challenge models to examine effectiveness of a feed additive or dietary strategy (80, 81). Such an *in vivo* model allows evaluation of a given feed additive in the context of an infectious pathogenic agent being part of the gastrointestinal ecosystem. However, in terms of identifying the most influential predisposing factors, a reliable and reproducible infection model is critical. Coccidia infection with live sporulated oocysts via oral gavage (crop) or litter contamination is commonly used in experimental coccidiosis models and is reasonably reliable in general (82). However, there are variation in experimental approaches related to dosing, species specification, timing and composition (wild-type or attenuated) for vaccination overdose and co-infection with *Clostridium perfringen* among others (82, 83). For nutrition research, it is imperative to have a coccidiosis model that is not only reproducible but target sections of the gut that have significant ramifications on nutrient digestion and absorption.

**Figure 1:**
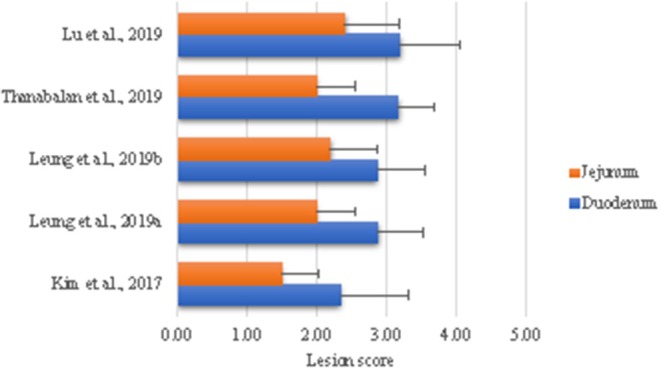
Intestinal lesion scores in broiler chicks challenged with *Eimeria*. All birds were challenged with 100,000 *E. acervullina* and 60,000 *E. maxima* sporulated oocysts on day 10 of age with exception of Kim et al. (84) in which case birds were challenged on day 5. Birds were necropsied 5 days post-challenge to assess lesion scores as described by Price et al. (90) using a scale of 0 (none) to 4 [high; Johnson and Reid (91)].

Our laboratory has developed a model to examine effects of coccidiosis on digestion and absorptive capacities and subsequent effects on GIT ecology. The rationale is to use this infection model to test dietary strategies during acute phase and recovery phases (84–88). The general approach is to challenge sub-samples of birds in a pen with high dose (100,000 *E. acervullina* and 60,000 *E. maxima* sporulated oocysts) to generate macroscopic lesions and the rest of birds with a low dose (25,000 *E. acervulina* and 5,000 *E. maxima* sporulated oocysts) in order to examine the consequences of altered nutrient digestion and absorption. Briefly, *Eimeria* parasites are propagated and purified according to Shirley (89) and dose is based on titration trials at Dr. John Barta's parasitology laboratory (University of Guelph). As shown in Figure 1, we have been able to reproduce consistent lesion scores in alignment with the biology of the challenge organisms, indicating high reproducibility of the challenge model. *Eimeria* destruction of intestinal lining results in poor growth performance, loose excreta and death in extreme cases (5, 14). The failure of parasitized animals to grow is partially due to loss of appetite and nutrient malabsorption (84, 86, 92–94). Structural and functional damages to the small intestine are indicated by the histomorphology, digestive enzymes, nutrients transporters, and nutrient retention in our model (84, 86, 87). Moreover, *E. acervulina* and *E. maxima* infection down regulates expression of digestive enzymes and nutrient transporters (84, 87, 93–95). Subsequent sections will evaluate literature where coccidiosis challenge was used to evaluate impact feed enzymes and yeast derivatives in modulating the expression of coccidiosis.

## Utility of Feed Enzymes in Modulating Coccidiosis in Broilers

**Figure 2:**
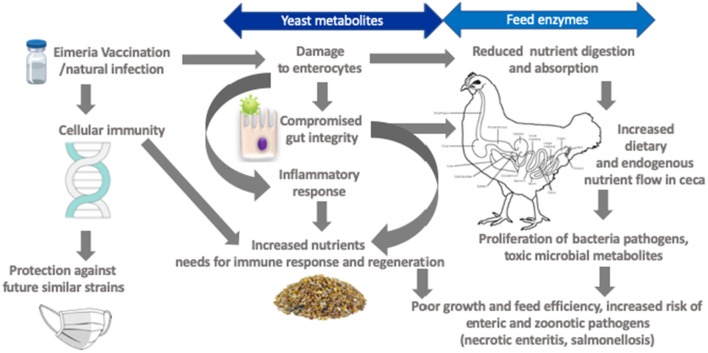
Framework for targets amenable by yeast derivatives and feed enzymes in mitigating negative effects of coccidiosis.

Coccidiosis effectively reduces nutrients digestion and absorption linked to anorexia in concurrence with morphological and functional intestinal damage (Figure 2) (84, 86, 87). Increased mucogenesis and enterocyte turnover (84), as well as post-absorptive metabolic changes and immune system activation, likely influence nutrient needs of broilers (9). For example, *Eimeria* infection increased proliferation of jejunal mucosal cells by 40% in concomitant with increased crypt depth indicating the birds prioritized gut development following intestinal insult (84). An increase in cell proliferation was also observed in the crypt base of *Eimeria* challenged chickens (96). In general, maintenance energy requirement increases in proportion to metabolic body size as the bird mature. However, it has been shown that coccidiosis markedly increased maintenance energy requirement. For example, Leung et al. (86), observed a 16% decrease in energy allocated to body weight gain (measured in caloric efficiency) in a 35-day old bird challenged with *E. acervulina* and *E. Maxima* at day 10 of age. The energy needed for immunity development was 5% in healthy birds compared to 28% for coccidiosis challenged birds and this cost became disproportionately elevated as the birds became heavier aged (97).

With diminution of digestive capacity and elevated inefficiency in energy utilization in coccidiosis afflicted birds; it is plausible that supplemental exogenous feed enzymes could be beneficial. However, few studies investigated the impact of supplementation of feed enzymes to complement endogenous enzymes (e.g., amylase, protease, lipase) in coccidiosis challenged birds. Dietary supplementation with a protease reduced negative impact of a coccidiosis infection (*E. acervulina, E. maxima*, and *E. tenella*) on body weight gain in broilers but had no effects on lesions and oocyst shedding (98). In contrast, Parker et al. (99) showed that an enzyme blend (amylase, protease, and xylanase) fed to coccidiosis vaccinated broiler chicks had no effect on ileal nutrient digestibility or growth performance but reduced lesion scores. *E. acervulina* and *E. maxima* associated intestinal damage have been linked with adverse effects on bone health mainly because they infect duodenum and upper jejunum, the major sites of minerals absorption (85, 100, 101). In this context, the role of supplemental phytase on mineral utilization has also been investigated in coccidia infection model (102, 103). The peculiarity is that the pH of duodenum of healthy birds is 6.0 or greater but is reduced to <5 in birds infected with coccidiosis (104). Lower duodenal pH is thought to enhance efficacy of phytase linked to optima pH range (2.5 and 5.5) for effective degradation of phytate (105). Indeed, phytase increased growth performance and tibia ash concentration in the presence or absence of *E. acervulina* (102). Coccidiosis reduced growth performance and absorption of calcium and phosphorous resulting in reduced bone strength, however, phytase supplementation did not mitigate negative effects of coccidiosis on phosphorous utilization (106). Supplementation of phytase, protease, and xylanase singly or in combination did not mitigate reduction in growth performance in broiler chickens exposed to a mixed coccidia vaccine (107).

It is important to consider relationship between *Eimeria* and *Clostridium perfringens* in evaluation of the role of feed enzymes in coccidiosis control*. Eimeria* infection increases endogenous losses of plasma proteins and mucin that nourishes *C. perfringens* (9). The large flow of nitrogenous materials in the ceca promotes production of toxic metabolites for example, thiols, amines, ammonia, and indoles (108), but most importantly high ceca digesta pH promotes proliferation of pathogens such as *C. perfringens* (9, 12). Feed enzymes can modulate GIT ecology reducing undigested nutrients and production of oligosaccharides with potential prebiotic effects (Figure 2) (24). For example, enzyme blend (amylase, protease, and xylanase) supplementation supported gut ecology that reduced intestinal lesion scores particularly in the ceca linked to altered microbial profiles in coccidia-vaccinated broilers (99). The authors interpreted that, although the enzyme blend did not influence ileal digestibility of nutrients it altered the characteristics of digesta such that ceca microbiota communities were altered. Phytase supplementation had no effects on oocyst shedding in naïve and coccidia vaccinated broilers subjected to *Eimeria* challenge but it reduced lesion scores (106). The effect of supplemental enzymes on lesion scores in coccidiosis afflicted broilers has been attributed to production of volatile fatty acids (34). An elegant study by Ruff et al. demonstrated that coccidiosis lowered luminal small intestine pH but increased ceca pH (109). It is therefore relevant that supplemental feed enzymes have been shown to increase ceca concentration of volatile fatty acids such as acetic and butyric acids with concomitant reduction of ceca digesta pH (29). Acetic acid was shown to be commensurate to anticoccidial drug (Amprolium) in suppressing coccidiosis associated negative effects on growth performance (110). Collectively, these studies suggested that supplemental enzymes somewhat affect survivability or extent of intestinal damage.

## Utility of Yeast Probiotics in Modulating Coccidiosis in Broilers

There are limited studies on yeast probiotic (e.g., *S. boulardii)* supplementation in broilers (111, 112). The common approach is a blend of yeast probiotics with bacterial cultures on the premise that beneficial effects of probiotics are genus, species and strain specific and use of multi-strain and multi-species might be more effective than mono-strain probiotics (54, 55). Indeed, some investigations have shown that co-supplementing yeast and bacterial probiotics enhanced their survival and growth (113, 114). Moreover, aggregation of *lactobacillus* with yeasts enhanced tolerance in gastric or intestinal juices (115). For example, a supplement containing *Lactobacillus acidophilus, Bacillus subtilis, S. cerevisiae*, and *A. oryzae* improved live body weight gain linked to enhanced nutrients utilization and intestinal microbial modulation (55). A combination of yeast and (*L. acidophilus* and *Streptococcus faecium*) enhanced growth performance in broilers through increased digestion and absorption of nutrients (116). *Lactobacillus fermentum* and *S. cerevisiae* was shown to modulate intestinal immune system without negative effects on growth performance in broilers (57). In contrast, a blend (*Lactobacillus plantarum, Lactobacillus delbrueckii* ssp. *bulgaricus, L. acidophilus, Lactobacillus rhamnosus, Bifidobacterium bifidum*, S*treptococcus salivarius* ssp*. thermophilus, Enterococcus faecium, A. oryzae*, and *Candida pintolepesii*) did not ameliorate negative effects of delayed feed access in newly hatched chicks on growth performance and gastrointestinal physiology (54). A challenge of evaluating studies of probiotic blends is that experimental design does not always incorporate single strains to characterize responses of each strain vs. combination.

*Saccharomyces cerevisiae* var*. boulardii* is one of the most researched non-bacterial probiotics with proven benefits in various human gastrointestinal disease models (117). This yeast was originally isolated from litchi fruit in Indochina by Henri Boulard in 1920 and has been used for treatment of intestinal diseases in children and adults since the 1950's (117, 118). Several mechanisms have been suggested as to the broad health-promoting effects of consuming yeast probiotics in humans and span from local general trophic effects to action on both innate and/or adaptive immunity (117, 119, 120). Clinical trials included mitigation of antibiotic-associated diarrhea; *Clostridium difficile* diarrhea, irritable bowel syndrome and inflammatory bowel diseases (117, 121). Folignè et al. (117) tested six yeast strains for anti-inflammatory potential and demonstrated that yeast-mediated protection seems to take place predominantly at the level of the intestinal mucosa. The authors indicated that prophylactic reinforcement and therapeutic restoration of barrier function by changing the luminal environment stimulated the mucosal barrier. This work extended previous observations that showed yeast probiotics enhanced epithelial integrity and reduced bacterial translocation in various sepsis models (122, 123). The mode of action of yeasts in controlling enteric diseases have not been elucidated but have been associated with release of antimicrobial peptides, acidification of surrounding environment, alteration of inflammatory and immune responses or destruction of toxic factors (63).

Although the use of probiotic yeast to control enteric diseases in humans has been studied extensively, little attention has been given to enteric diseases of farm animals. In one study, broiler chicks were fed 1 or 100 g *S. boulardii*/kg feed and challenged with *S. typhimurium* (124). The authors observed 70% colonization in the ceca of control bird vs. 20 or 5% colonization in birds fed 1 or 100 g, respectively. One innovative study investigated the anticoccidial activity of a compound (s) isolated from *Meyerozyma guilliermondii* yeast culture (125). The compounds were shown to have anticoccidial activity against *E. tenella* oocysts under *in vitro* simulation linked to reduced oocysts viability. In other investigations, *S. cerevisiae* was investigated as oral *Eimeria* vaccine delivery vehicle commensurate to previous successful delivery of oral viral vaccines (126, 127). In this context, a microneme protein (EtMic2) of *E. tenella* that is intimately involved in host-cell invasion (128) was expressed on the surface of live *S. cerevisiae* cells (129). The whole yeast cells without or with EtMic2 on their surfaces was used as a live oral vaccine against *E. tenella* challenge in pullets. Significantly lower oocyst shedding, and lesion scores were observed in birds receiving EtMic2 yeast relative to the control or the birds receiving yeast without EtMic2 protein (129). The EtMic2 group also showed higher weight gains. In general, the utility of coccidiosis vaccination in the industry mainly relies on development of cellular immunity for protection against later *Eimeria* exposure (130). However, immunodominant surface antigens identified in *E. acervulina* and *E. maxima* have been shown to elicit measurable antibody responses particularly production of IgA in addition to stimulating cell-mediated immunity (131). Therefore, there is potential for antibodies (raised by live immunization or against purified stage-specific *Eimeria* antigens) to inhibit parasite development (128, 129, 132). Field applications of anticoccidial compounds secreted by yeasts and/or utility of yeast to deliver vaccines need to be investigated for commercial application as complement for anticoccidial drugs and/or as adjuvant for cocci vaccines.

## Utility of Nucleotide Rich Yeast Extracts in Modulating Coccidiosis in Broilers

As basic units of the DNA and RNA, nucleotides are present in all living cells (133, 134). With adequate supply of energy and amino acids, nucleotides can be synthesized *de novo*. However, they may become conditionally essential during illness, periods of limited feed intake or rapid growth (133, 134). These conditions are commensurate to gastrointestinal damage occasioned by *Eimeria* infection (9). Evaluations of nucleotide rich yeast extracts on growth, feed efficiency and intestinal development and health has been reported in broiler chickens (86, 87, 135, 136). However, there are limited studies assessing the effects of dietary nucleotide supplementation on development of immune organs in broilers challenged with *Eimeria*. Human infants fed nucleotides were shown to have increased production of IgA (137) linked to the requirement of lymphocytes for exogenous nucleotides (138). Moreover, dietary nucleotides supplementation in infants have been shown to affect other immune functions such as the activation of NK cells and macrophages, the production of splenic cytokines, and the number of antibody secreting cells (138). Leung et al. (87) evaluated the effect of supplementation of nucleotide rich yeast nucleotide supplement on growth performance, intestinal histomorphology, expression of select intestinal genes and microbial activity during the acute phase of *Eimeria* challenge (7 d post-challenge). Supplemental nucleotides improved jejunal histomorphology and expression of the nutrient transporter cationic amino acid transporter 1 (CAT1). Interestingly, the impact of nucleotides and *Eimeria* were independent on microbiota community but interactive on microbial activity. Instructively, there was a trend for decreasing alpha diversity with nucleotide supplementation commensurate to that seen with provision of antimicrobials (139). These changes on alpha diversity and in microbiota populations may have long-term impacts or may be further amplified with time. Effects could also cascade down into production of volatile fatty acids and change the cecal pH and subsequently factors such as histomorphology and immune system development. In further studies, Leung et al. (86) fed broiler chickens nucleotides rich yeast extract and challenged them with *Eimeria* on d 10 post-hatch. The concentration of plasma and mucosal IgA and immune organ weights (bursa, spleen, and thymus) were determined at d 5 and 25 post-challenge. There were no effects on d 5 post-challenge measurements, however, birds fed yeast nucleotides showed heavier bursa on d 25 post-challenge. It seems that nucleotides supplementation can attenuate some of the negative effects of *Eimeria*, however, further investigations are warranted to determine the optimal supplementation period and concentration and the effect of individual nucleotides.

## Utility of Yeast Cell Wall (YCW) Components in Modulating Coccidiosis in Broilers

Yeast cell wall components (β-glucans and mannans) have been linked to modulation of immune system (140, 141), binding to toxins, and to pathogenic cells (142), and interactions with gut constituents (143, 144). Hooge (145), reported a meta-analyses of *S. cerevisiae* var*. boulardii* cell wall components supplemented diets vs. negative control (antibiotic free, 29 experiments) or antibiotic supplemented positive control (antimicrobial growth promoter, 21 experiments) diets. The meta-analyses revealed small magnitude (<2% improvement vs. negative control) but significant impact of YCW on body weight gain and feed conversion ratio but no difference between positive control (145). However, birds fed YCW showed 21.4 and 18.1% lower mortality compared with negative and positive control, respectively. Yeast cell wall products can serve as microbe-associated molecular patterns and modulate the expression of pattern recognition receptors (PRRS) (136, 146). Indeed, supplemental YCW showed commensurate growth performance and livability responses to zinc bacitracin and Salinomycin in a *Eimeria* and *C. perfringens* co-infection model (147). Chicken macrophages are involved the adaptive immune responses through interaction with *Eimeria* in the intestinal mucosa (148). Immunodominant surface antigens identified in *E. acervulina* and *E. maxima* have been shown to elicit measurable antibody responses with IgA being the most important isotype (131). Thus, although *E. acervulina* and *E. maxima* challenge reduced jejunal mucosa IgA by 33% at 5 days post-challenge, the concentration was increased by 16% at 25 days post-challenge (86). A further study indicated that *E. acervulina* and *E. maxima* challenge increased jejunal IgA by 64% at 5 days post-challenge (149). Although specific mechanisms of action intestinal IgA on coccidial infections are still subject of investigations, it has been speculated that IgA reduces development of sporozoites or merozoites and prevent host cell invasion (150, 151). The relevance is that YCW components are known to be immunomodulators and it has been demonstrated that that dietary supplementation increases local mucosal IgA secretions as well as cellular and humoral immune responses (Figure 2) (152).

It is plausible YCW components can modulate cellular and humoral mediated immune responses against coccidial infections (130). Dietary YCW (1 or 10 g/kg) reduced severity of infection and oocyst shedding from a single *E. tenella* or mixture of *E. acervulina, E. maxima* and *E. tenella* challenge in broiler chickens (153, 154). Dietary supplementation with 5 g/kg of autolyzed *S. cerevisiae* derivatives stimulated intestinal mucosal IgA secretion, humoral, and cell-mediated immune responses, and reduced oocysts shedding in broilers subjected to coccidia vaccination (152). However, YCW (0.5 g/kg) fed singly or in combination with tannin (0.5 g/kg) did not reduced severity of infection with a mixture of *E. acervulina, E. maxima*, and *E. tenella* in broilers. Broilers fed a supplement (1–2 g/kg) containing whole dead yeast exhibited decrease in oocysts shedding as well as increased macrophage nitric oxide production and inflammatory cytokine production (155). A refined mixture of YCW and β-glucans and a crude yeast extract were tested in broilers subjected to 10 times Coccivac B vaccine (156). Although both products had no impact on growth performance relative to the control, birds fed refined mixture had lower expression of IL-6 in the ileal mucosa and those fed crude yeast showed improved serum immunoglobulin G (156). These contradictory results are possibly linked to the differences in YCW inclusion in the feed and doses of *Eimeria* spp. inoculation.

Investigations in developmental programming has added to our understanding of the maternal offspring interface and continues to raise important questions regarding nutritional management of breeding animals (157, 158). In avian species, immuno-competence development is initiated during the embryogenesis (159). Protective role of maternal antibodies is critical because of precocial nature of avian species (33). The breeder antibodies are deposited in the egg and continue to function in early life of chicks (33). The role of maternal immunity on coccidial infections in chicks has been investigated (160, 161). For example, infection of breeder hens with *Eimeria maxima* induced production of parasite-specific antibodies which were transferred to chicks (160). These antibodies were highly protective, mediating up to a 97% reduction in oocyst shedding in challenged hatchlings. We recently showed that feeding broiler breeders yeast product rich in enzyme hydrolyzed cell wall components increased deposition of IgA in the hatching egg yolks (149). The data further indicated that feeding hydrolyzed yeast cell wall to broiler breeders and to the chicks improved jejunal histomorphology independent of *Eimeria* challenge (149).

## Further Refinement and Future Directions

The global poultry production has tripled to an annual production of 90 million tons of chicken meat and 1.1 trillion eggs (http://www.fao.org/faostat/) in the last two decades. Further expansion is expected in response to burgeoning human population. The sector is also under pressure to produce food products in ways that are ethical, environmentally sustainable, and wholesome. For example, indiscriminate use of antibiotics is a topical global issue with increasing emphasis on alternative strategies for effective prevention and control of enteric pathogens. Herein is an overview of coccidiosis, its implications on intestinal health and function and targets amenable to modulation by feed enzymes and yeast derivatives (Figure 2). To a large extent the poultry industry has widely accepted the use of feed enzymes to improve feed digestion. It is plausible that supplemental exogenous feed enzymes could counteract diminution of digestive capacity in coccidiosis afflicted birds. Feed enzymes could modulate ceca ecology by reducing the flow of undigested nutrients and promoting acidic fermentation. Effectively creating conditions that reduce survivability of *Eimeria* and *C. perfringens* proliferation. Yeast and yeast derivatives have been associated with alteration of inflammatory and immune responses. There is tremendous opportunity for developing new generation of yeast derivatives with optimized features in terms of interaction with *Eimeria* and modulation of the host immune system. The protective role of maternal antibodies is of interest because the antibodies deposited in the egg and the levels transferred to the offspring are directly related to the circulating levels of these immunoglobulin in the dam. More research is needed to refine the relationship between composition and function of yeast derivatives with a view of selecting new generation of yeast fractions with optimized characteristics for application in broiler breeders. Like many feed additives evaluations, inconsistencies of responses are often a major concern. Utility of experimental disease challenge model can help to mimicry field conditions; however, correct dosing of active ingredient is critical. Although suppliers and regulatory agencies have advanced use of feed enzymes to the extent dosage and recovery in the feed can be determined, there is paucity on analytics for quantifying yeast derivatives in-feed which is critical for accurate dosing. Accurate dosing is essential for achieving desired benefits and preventing excessive feeding of biologically active components. Moreover, feed additives research rarely considers commercial broiler feed contains a range of additives. Perhaps, future feed additives investigations should consider co-supplementation of to document responses and potential synergies.

## Author Contributions

HL and RA were graduate students of EK and were involved in execution of coccidiosis challenge studies and some literature search. RP provided technical knowhow on feed enzymes and yeast derivatives and reviewed integration of concepts. JB is an expert parasitologist on *Eimeria* challenge model. All authors reviewed the manuscript. EK searched literature and wrote significant portion of the review and had overall conceptual and editorial responsibility.

### Conflict of Interest

RP is an employee of Canadian Bio-Systems Inc. The remaining authors declare that the research was conducted in the absence of any commercial or financial relationships that could be construed as a potential conflict of interest.

## References

[1] Mottet A Tempio G Global poultry production: current state and future outlook and challenges. World Poult Sci J. (2017) 73:245–56. 10.1017/S0043933917000071

[2] Dalloul RA Lillehoj HS. Poultry coccidiosis: recent advancements in control measures and vaccine development. Expert Rev Vacc. (2006) 5:143–63. 10.1586/14760584.5.1.143 16451116

[3] Kadykalo S Roberts T Thompson M Wilson J Lang M Espeisse O. The value of anticoccidials for sustainable global poultry production. Int J Antimicrob Agents. (2018) 51:304–10. 10.1016/j.ijantimicag.2017.09.004 28935212

[4] Barta JR Coccidiosis. In: eLS. New York, NY: John Wiley and Sons (2001). 10.1038/npg.els.0001947

[5] Chapman HD. Milestones in avian coccidiosis research: a review. Poult Sci. (2014) 93:501–11. 10.3382/ps.2013-03634 24604841

[6] Chapman HD Barta JR Hafeez MA Matsler P Rathinam T Raccoursier M. The epizootiology of *Eimeria* infections in commercial broiler chickens where anticoccidial drug programs were employed in six successive flocks to control coccidiosis. Poult Sci. (2016) 95:1774–8. 10.3382/ps/pew091 27053624

[7] Chapman HD Roberts B Shirley MW Williams RB. Guidelines for evaluating the efficacy and safety of live anticoccidial vaccines, and obtaining approval for their use in chickens and turkeys. Avian Pathol. (2005) 34:279–90. 10.1080/03079450500178378 16147563

[8] Shirley MW Smith AL Tomley FM. The biology of Avian *Eimeria* with an emphasis on their control by vaccination. In: Baker JR Muller R Rollinson D editors. Advances in Parasitology. Amsterdam: Academic Press (2005), p. 285–330. 10.1016/S0065-308X(05)60005-X 16230106

[9] Williams RB. Intercurrent coccidiosis and necrotic enteritis of chickens: rational, integrated disease management by maintenance of gut integrity. Avian Pathol. (2005) 34:159–80. 10.1080/03079450500112195 16191699

[10] Arakawa A Baba E Fukata T. *Eimeria tenella* infection enhances *Salmonella typhimurium* infection in chickens. Poult Sci. (1981) 60:2203–9. 10.3382/ps.0602203 7329903

[11] Williams RB. Anticoccidial vaccines for broiler chickens: pathways to success. Avian Pathol. (2002) 31:317–53. 10.1080/03079450220148988 12396335

[12] Timbermont L Haesebrouck F Ducatelle R Van Immerseel F. Necrotic enteritis in broilers: an updated review on the pathogenesis. Avian Pathol. (2011) 40:341–7. 10.1080/03079457.2011.590967 21812711

[13] Cosby DE Cox NA Harrison MA Wilson JL Buhr RJ Fedorka-Cray PJ Salmonella and antimicrobial resistance in broilers: a review. J Appl Poult Res. (2015) 24:408–26. 10.3382/japr/pfv038

[14] Chapman HD. A landmark contribution to poultry science–prophylactic control of coccidiosis in poultry. Poult Sci. (2009) 88:813–5. 10.3382/ps.2008-00316 19276426

[15] Chapman HD Jeffers TK. Vaccination of chickens against coccidiosis ameliorates drug resistance in commercial poultry production. Int J Parasitol. (2014) 4:214–7. 10.1016/j.ijpddr.2014.10.002 25516830 PMC4266793

[16] Joyner LP. Immunological variation between two strains of *Eimeria acervulina*. Parasitology. (1969) 59:725–73. 10.1017/S0031182000031243 5374708

[17] Martin AG Danforth HD Barta JR Fernando MA. Analysis of immunological cross-protection and sensitivities to anticoccidial drugs among five geographical and temporal strains of *Eimeria maxima*. Int. J. Parasitol. (1997) 27:527–33. 10.1016/S0020-7519(97)00027-1 9193946

[18] Kogut MH Klasing K An immunologist's perspective on nutrition, immunity, and infectious diseases: introduction and overview. J Appl Poult Res. (2009) 18:103–10. 10.3382/japr.2008-00080

[19] Quiroz-Castañeda RE Dantán-González E. Control of avian coccidiosis: future and present natural alternatives. Biomed Res Int. (2015) 2015:430610. 10.1155/2015/430610 25785269 PMC4346696

[20] Hervey GW. A nutritional study upon a fungus enzyme. Science. (1925) 62:247. 10.1126/science.62.1602.247 17752315

[21] Masey O'neill HV Smith JA Bedford MR Multicarbohydrase enzymes for non-ruminants. Asian-australas. J Anim Sci. (2014) 27:290–301. 10.5713/ajas.2013.13261 PMC4093217 25049954

[22] Bedford MR Schulze H. Exogenous enzymes for pigs and poultry. Nutr Res Rev. (1998) 11:91–114. 10.1079/NRR19980007 19087461

[23] Slominski BA. Recent advances in research on enzymes for poultry diets. Poult Sci. (2011) 90:2013–23. 10.3382/ps.2011-01372 21844268

[24] Kiarie E Romero LF Nyachoti CM. The role of added feed enzymes in promoting gut health in swine and poultry. Nutr Res Rev. (2013) 26:71–88. 10.1017/S0954422413000048 23639548

[25] Kiarie E Walsh MC Nyachoti CM Performance, digestive function, and mucosal responses to selected feed additives for pigs. J Anim Sci. (2016) 94:169–80. 10.2527/jas.2015-9835

[26] Kiarie EG Mills A. Role of feed processing on gut health and function in pigs and poultry: conundrum of optimal particle size and hydrothermal regimens. Front Vet Sci. (2019) 6:19. 10.3389/fvets.2019.00019 30838217 PMC6390496

[27] Adeola O Cowieson AJ. Board-invited review: opportunities and challenges in using exogenous enzymes to improve nonruminant animal production. J Anim Sci. (2011) 89:3189–218. 10.2527/jas.2010-3715 21512114

[28] Ravindran V Advances and future directions in poultry nutrition: an overview. Korean J Poult Sci. (2012) 39:53–62. 10.5536/KJPS.2012.39.1.053

[29] Kiarie E Romero LF Ravindran V Growth performance, nutrient utilization, and digesta characteristics in broiler chickens fed corn or wheat diets without or with supplemental xylanase. Poult Sci. (2014) 93:1186–96. 10.3382/ps.2013-03715 24795311

[30] Kiarie E Walsh MC Romero LF Arent S Ravindran V. Nutrient and fiber utilization responses of supplemental xylanase in broiler chickens fed wheat based diets are independent of the adaptation period to test diets. Poult Sci. (2017) 96:3239–45. 10.3382/ps/pex100 28419372

[31] Croom WJ Brake J Coles BA Havenstein GB Christensen VL Mcbride BW Is intestinal absorption capacity rate-limiting for performance in poultry? J Appl Poult Res. (1999) 8:242–52. 10.1093/japr/8.2.242

[32] Gilbert ER Li H Ernmersonj DA Webb KE Wong EA. Developmental regulation of nutrient transporter and enzyme mRNA abundance in the small intestine of broilers. Poult Sci. (2007) 86:1739–53. 10.1093/ps/86.8.1739 17626820

[33] Friedman A Elad O Cohen I Bar Shira E The gut associated lymphoid system in the post-hatch chick: dynamics of maternal IgA. Isr J Vet Med. (2012) 67:75–81. Available online at: http://www.ijvm.org.il/sites/default/files/friedman.pdf

[34] Apajalahti J Kettunen A Graham H Characteristics of the gastrointestinal microbial communities, with special reference to the chicken. World Poult Sci J. (2004) 60:223–32. 10.1079/WPS20040017

[35] Courtin CM Broekaert WF Swennen K Lescroart O Onagbesan O Buyse J Dietary inclusion of wheat bran arabinoxylooligosaccharides induces beneficial nutritional effects in chickens. Cereal Chem. (2008) 85:607–13. 10.1094/CCHEM-85-5-0607

[36] Wealleans AL Walsh MC Romero LF Ravindran V. Comparative effects of two multi-enzyme combinations and a Bacillus probiotic on growth performance, digestibility of energy and nutrients, disappearance of non-starch polysaccharides, and gut microflora in broiler chickens. Poult Sci. (2017) 96:4287–97. 10.3382/ps/pex226 29053809 PMC5850647

[37] Bedford MR. The evolution and application of enzymes in the animal feed industry: the role of data interpretation. Br Poult Sci. (2018) 59:486–93. 10.1080/00071668.2018.1484074 29877713

[38] Bennett JW Mycotechnology: the role of fungi in biotechnology1Based on a lecture held at the symposium, ‘Progress in US Biotechnology', at the 8th European Congress on Biotechnology (ECB8) in Budapest, Hungary, August 1997.1. J Biotechnol. (1998) 66:101–7. 10.1016/S0168-1656(98)00133-3 9866863

[39] Barnett JA. A history of research on yeasts 2: Louis Pasteur and his contemporaries, 1850–1880. Yeast. (2000) 16:755–71. 10.1002/1097-0061(20000615)16:8<755::AID-YEA587>3.0.CO;2-4 10861901

[40] Kurtzman C Fell JW Boekhout T Classification of yeast. In: Kurtzman C Fell JW Boekhout T editors. The Yeasts, A Taxonomic Study. London: Elsevier (2011), p. 3–8. 10.1016/B978-0-444-52149-1.00001-X

[41] Jansen MLA Bracher JM Papapetridis I Verhoeven MD De Bruijn H De Waal PP. *Saccharomyces cerevisiae* strains for second-generation ethanol production: from academic exploration to industrial implementation. FEMS Yeast Res. (2017) 17:fox044. 10.1093/femsyr/fox044 28899031 PMC5812533

[42] Nielsen J Larsson C Van Maris A Pronk J. Metabolic engineering of yeast for production of fuels and chemicals. Curr Opin Biotechnol. (2013) 24:398–404. 10.1016/j.copbio.2013.03.023 23611565

[43] Shurson GC Yeast and yeast derivatives in feed additives and ingredients: sources, characteristics, animal responses, and quantification methods. Anim Feed Sci Technol. (2018) 235:60–76. 10.1016/j.anifeedsci.2017.11.010

[44] Stein HH Shurson GC. Board-invited review: the use and application of distillers dried grains with solubles in swine diets. J Anim Sci. (2009) 87:1292–303. 10.2527/jas.2008-1290 19028847

[45] Shurson GC. The Role of Biofuels Coproducts in Feeding the World Sustainably. Ann Rev Anim Biosci. (2017) 5:229–54. 10.1146/annurev-animal-022516-022907 27813679

[46] Ugalde UO Castrillo JI Single cell proteins from fungi and yeasts. In: Khachatourians GG Arora DK editors. Applied Mycology and Biotechnology. Amsterdam: Elsevier (2002), p. 123–49. 10.1016/S1874-5334(02)80008-9

[47] Kollár R Reinhold BB Petráková E Yeh HJC Ashwell G Drgonová J. Architecture of the yeast cell wall: β(1 → 6)-glucan interconnects mannoprotein, β(1 → 3)-glucan, and chitin. J Biol Chem. (1997) 272:17762–75. 10.1074/jbc.272.28.17762 9211929

[48] Cabib E Farkas V Kosík O Blanco N Arroyo J Mcphie P. Assembly of the yeast cell wall: Crh1p and Crh2p act as transglycosylases *in vivo* and *in vitro*. J Biol Chem. (2008) 283:29859–72. 10.1074/jbc.M804274200 18694928 PMC2573080

[49] Waititu SM Yitbarek A Matini E Echeverry H Kiarie E Rodriguez-Lecompte JC. Effect of supplementing direct-fed microbials on broiler performance, nutrient digestibilities, and immune responses. Poult Sci. (2014) 93:625–35. 10.3382/ps.2013-03575 24604856

[50] Mohammadigheisar M Shirley RB Barton J Welsher A Thiery P Kiarie E. Growth performance and gastrointestinal responses in heavy Tom turkeys fed antibiotic free corn-soybean meal diets supplemented with multiple doses of a single strain *Bacillus subtilis* probiotic (DSM29784). Poult Sci. (2019) 98:5541–50. 10.3382/ps/pez305 31180117

[51] Neijat M Habtewold J Shirley RB Welsher A Barton J Thiery P *Bacillus subtilis* DSM29784 modulates cecal microbiome, short chain fatty acids concentration, and apparent retention of dietary components in Shaver Whites during grower, developer and laying phases. Appl Environ Microbiol. (2019) 85:e00402–19. 10.1128/AEM.00402-19 31076425 PMC6606875

[52] Neijat M Kiarie E Welsher A Barton J Shirley RB Thiery P. Growth performance, apparent retention of components, and excreta dry matter content in Shaver White pullets (5 to 16 week of age) in response to dietary supplementation of graded levels of a single strain *Bacillus subtilis* probiotic. Poult Sci. (2019) 98:3777–86. 10.3382/ps/pez080 30839091

[53] Neijat M Shirley RB Barton J Thiery P Welsher A Kiarie E. Effect of dietary supplementation of *Bacillus subtilis* DSM29784 on hen performance, egg quality indices, and apparent retention of dietary components in laying hens from 19 to 48 weeks of age. Poult Sci. (2019) 98:5622–35. 10.3382/ps/pez324 31222316

[54] Daşkiran M Önol AG Cengiz Ö Ünsal H Türkyilmaz S Tatli O Influence of dietary probiotic inclusion on growth performance, blood parameters, and intestinal microflora of male broiler chickens exposed to posthatch holding time. J Appl Poult Res. (2012) 21:612–22. 10.3382/japr.2011-00512

[55] Shim YH Ingale SL Kim JS Kim KH Seo DK Lee SC. A multi-microbe probiotic formulation processed at low and high drying temperatures: effects on growth performance, nutrient retention and caecal microbiology of broilers. Br Poult Sci. (2012) 53:482–90. 10.1080/00071668.2012.690508 23130583

[56] Rahman M Mustari A Salauddin M Rahman M Effects of probiotics and enzymes on growth performance and haematobiochemical parameters in broilers. J Bangl Agric Univ. (2013) 11:111–8. 10.3329/jbau.v11i1.18221

[57] Bai SP Wu AM Ding XM Lei Y Bai J Zhang KY. Effects of probiotic-supplemented diets on growth performance and intestinal immune characteristics of broiler chickens. Poult Sci. (2013) 92:663–70. 10.3382/ps.2012-02813 23436517

[58] Chaucheyras-Durand F Chevaux E Martin C Forano E Use of yeast probiotics in ruminants: effects and mechanisms of action on rumen pH, fibre degradation, and microbiota according to the diet. In: Rigobelo EC editor. Probiotic in Animals. IntechOpen (2012), p. 119–52. 10.5772/50192

[59] Vohra A Syal P Madan A Probiotic yeasts in livestock sector. Anim Feed Sci Technol. (2016) 219:31–47. 10.1016/j.anifeedsci.2016.05.019

[60] Bayrock D Ingledew WM Fluidized bed drying of baker's yeast: moisture levels, drying rates, and viability changes during drying. Food Res Int. (1997) 30:407–15. 10.1016/S0963-9969(98)00003-9

[61] Aguirre-Guzmán G Ricque-Marie D Cruz-Suárez LE Survival of agglomerated *Saccharomyces cerevisiae* in pelleted shrimp feeds. Aquaculture. (2002) 208:125–35. 10.1016/S0044-8486(01)00711-6

[62] Bayrock D Ingledew WM Mechanism of viability loss during fluidized bed drying of baker's yeast. Food Res Int. (1997) 30:417–25. 10.1016/S0963-9969(97)00072-0

[63] Hatoum R Labrie S Fliss I. Antimicrobial and probiotic properties of yeasts: from fundamental to novel applications. Front Microbiol. (2012) 3:421. 10.3389/fmicb.2012.00421 23267352 PMC3525881

[64] Payen A Persoz JF Mémoire sur la diastase, les principaux produits de ses réactions et leurs applications aux arts industriels [Memoir on diastase, the principal products of its reactions, and their applications to the industrial arts] [Online]. (1933). Available online at: https://en.wikipedia.org/wiki/Enzyme#cite_note-9 (accessed June 29, 2019).

[65] Manchester KL. Louis Pasteur (1822–1895)—chance and the prepared mind. Trends Biotechnol. (1995) 13:511–5. 10.1016/S0167-7799(00)89014-9 8595136

[66] Klabunde J Kunze G Gellissen G Hollenberg CP. Integration of heterologous genes in several yeast species using vectors containing a Hansenula polymorpha-derived rDNA-targeting element. FEMS Yeast Res. (2003) 4:185–93. 10.1016/S1567-1356(03)00148-X 14613883

[67] Schuller D Casal M. The use of genetically modified *Saccharomyces cerevisiae* strains in the wine industry. Appl Microbiol Biotechnol. (2005) 68:292–304. 10.1007/s00253-005-1994-2 15856224

[68] Johnson EA Biotechnology of non-saccharomyces yeasts—the ascomycetes. Appl Microbiol Biotechnol. (2013) 97:503–17. 10.1007/s00253-012-4497-y 23184219

[69] Kim H Yoo SJ Kang HA. Yeast synthetic biology for the production of recombinant therapeutic proteins. FEMS Yeast Res. (2015) 15:1–16. 10.1111/1567-1364.12195 25130199

[70] Waldron C Lacroute F. Effect of growth rate on the amounts of ribosomal and transfer ribonucleic acids in yeast. J Bacteriol. (1975) 122:855–65. 1097403 10.1128/jb.122.3.855-865.1975 PMC246135

[71] Běhalová B Bláhová M Šillinger V Machek F Comparison of various ways of extraction of nucleic acids and of preparation of yeast extract from *saccharomyces cerevisiae* and *Candida utilis*. Acta Biotechnol. (1991) 11:547–52. 10.1002/abio.370110608

[72] Chaffin WL López-Ribot JL Casanova M Gozalbo D Martínez JP. Cell wall and secreted proteins of *Candida albicans*: identification, function, and expression. Microbiol Mol Biol Rev. (1998) 62:130–80. 9529890 10.1128/mmbr.62.1.130-180.1998 PMC98909

[73] Chew LY Toh GT Ismail A Chapter 15 - application of proteases for the production of bioactive peptides. In Kuddus M editor. Enzymes in Food Biotechnology, Amsterdam: Academic Press (2019), p. 247–61. 10.1016/B978-0-12-813280-7.00015-3

[74] Kempler GM. Production of flavor compounds by microorganisms. In: Laskin AI editors. Advances in Applied Microbiology. Academic Press (1983), p. 29–51. 10.1016/S0065-2164(08)70353-8 6650264

[75] Aimanianda V Clavaud C Simenel C Fontaine T Delepierre M Latgé J-P. Cell wall beta-(1,6)-glucan of *Saccharomyces cerevisiae*: structural characterization and *in situ* synthesis. J Biol Chem. (2009) 284:13401–12. 10.1074/jbc.M807667200 19279004 PMC2679440

[76] Goodridge HS Wolf AJ Underhill DM. Beta-glucan recognition by the innate immune system. Immunol Rev. (2009) 230:38–50. 10.1111/j.1600-065X.2009.00793.x 19594628 PMC6618291

[77] Bzducha-Wróbel A Błazejak S Kawarska A Stasiak-Rózanska L Gientka I Majewska E. Evaluation of the efficiency of different disruption methods on yeast cell wall preparation for β-glucan isolation. Molecules. (2014) 19:20941–61. 10.3390/molecules191220941 25517337 PMC6271764

[78] Schiavone M Vax A Formosa C Martin-Yken H Dague E François JM. A combined chemical and enzymatic method to determine quantitatively the polysaccharide components in the cell wall of yeasts. FEMS Yeast Res. (2014) 14:933–47. 10.1111/1567-1364.12182 25041403

[79] Lesage G Bussey H. Cell wall assembly in *Saccharomyces cerevisiae*. Microbiol Mol Biol Rev. (2006) 70:317–43. 10.1128/MMBR.00038-05 16760306 PMC1489534

[80] Cooper KK Songer JG. Virulence of *Clostridium perfringens* in an experimental model of poultry necrotic enteritis. Vet Microbiol. (2010) 142:323–8. 10.1016/j.vetmic.2009.09.065 19931323

[81] Peek HW Landman WJM. Coccidiosis in poultry: anticoccidial products, vaccines and other prevention strategies. Vet Q. (2011) 31:143–61. 10.1080/01652176.2011.605247 22029884

[82] Elmusharaf MA Mohamed HE Alhaidary A Beynen AC Efficacy and characteristics of different methods of coccidiosis infection in broiler chickens. Am J Anim Vet Sci. (2010) 5:45–50. 10.3844/ajavsp.2010.45.51

[83] Shojadoost B Vince AR Prescott JF. The successful experimental induction of necrotic enteritis in chickens by *Clostridium perfringens*: a critical review. Vet Res. (2012) 43:74. 10.1186/1297-9716-43-74 23101966 PMC3546943

[84] Kim E Leung H Akhtar N Li J Barta JR Wang Y. Growth performance and gastrointestinal responses of broiler chickens fed corn-soybean meal diet without or with exogenous epidermal growth factor upon challenge with *Eimeria*. Poult Sci. (2017) 96:3676–86. 10.3382/ps/pex192 28938785 PMC5850350

[85] Akbari Moghaddam Kakhki R Lu Z Thanabalan A Leung H Mohammadigheisar M Kiarie E. *Eimeria* challenge adversely affected long bone attributes linked to increased resorption in 14-day-old broiler chickens. Poult Sci. (2019) 98:1615–21. 10.3382/ps/pey527 30544238 PMC6414031

[86] Leung H Patterson R Barta JR Karrow N Kiarie E Nucleotide-rich yeast extract fed to broiler chickens challenged with *Eimeria*: impact on growth performance, jejunal histomorphology, immune system, and apparent retention of dietary components and caloric efficiency. Poult Sci. (2019) 98:4375–83. 10.3382/ps/pez213 31329966

[87] Leung H Yitbarek A Snyder R Patterson R Barta JR Karrow N Responses of broiler chickens to *Eimeria* challenge when fed a nucleotide-rich yeast extract. Poult Sci. (2019) 98:1622–33. 10.3382/ps/pey533 30481335 PMC6414034

[88] Thanabalan A Moats J Price K Kiarie E Omega-3 fatty acids sources fed to broiler breeders and/or their progeny: impact on growth performance and breast yield in 42-day old broiler chickens. In: 2019 Poultry Science Association Annual Meeting. Montreal, QC: Poultry Science Association (2019).

[89] Shirley MW *Eimeria* species and strains in chickens. In: Eckert J Braun R Shirley MW Coudert P editors. COST89/820, Biotechnology Guidelines on Techniques in Coccidiosis Research. Luxembourg: European Commission (1995), p. 1–25.

[90] Price KR Guerin MT Barta JR Success and failure: the role of relative humidity levels and environmental management in live Eimeria vaccination of cage-reared replacement layer pullets. J. Appl. Poultry Res. (2014) 23:523–35. 10.3382/japr.2014-00989

[91] Johnson J Reid M. Anticoccidial drugs: lesion scoring techniques in battery and floor-pen experiments with chickens. Exp Parasitol. (1970) 28:30–6. 10.1016/0014-4894(70)90063-9 5459870

[92] Major JR Jr Ruff MD. *Eimeria* spp.: influence of coccidia on digestion (amylolytic activity) in broiler chickens. Exp Parasitol. (1978) 45:234–40. 10.1016/0014-4894(78)90064-4 680079

[93] Su S Miska KB Fetterer RH Jenkins MC Wong EA. Expression of digestive enzymes and nutrient transporters in Eimeria acervulina-challenged layers and broilers. Poult Sci. (2014) 93:1217–26. 10.3382/ps.2013-03807 24795315

[94] Su S Miska KB Fetterer RH Jenkins MC Wong EA. Expression of digestive enzymes and nutrient transporters in *Eimeria*-challenged broilers. Exp Parasitol. (2015) 150:13–21. 10.1016/j.exppara.2015.01.003 25617757

[95] Adams C Vahl HA Veldman A. Interaction between nutrition and Eimeria acervulina infection in broiler chickens: development of an experimental infection model. Br J Nutr. (1996) 75:867–73. 10.1079/BJN19960192 8774231

[96] Sun L Dong H Zhang Z Liu J Hu Y Ni Y. Activation of epithelial proliferation induced by *Eimeria acervulina* infection in the duodenum may be associated with cholesterol metabolism. Oncotarget. (2016) 7:27627–40. 10.18632/oncotarget.8490 27050279 PMC5053676

[97] Teeter RG Beker A How management, intestinal health influence poultry calorific efficiency. In: WATT Poultry USA. Rockford, IL: WATT Global Media (2011). Available online at: https://www.wattagnet.com/articles/11083-how-management-intestinal-health-influence-poultry-caloric-efficiency

[98] Peek HW Van Der Klis JD Vermeulen B Landman WJM Dietary protease can alleviate negative effects of a coccidiosis infection on production performance in broiler chickens. Anim Feed Sci Technol. (2009) 150:151–9. 10.1016/j.anifeedsci.2008.08.006

[99] Parker J Oviedo-Rondón EO Clack BA Clemente-Hernández S Osborne J Remus JC. Enzymes as feed additive to aid in responses against eimeria species in coccidia-vaccinated broilers fed corn-soybean meal diets with different protein levels. Poult Sci. (2007) 86:643–53. 10.1093/ps/86.4.643 17369534

[100] Turk DE. Calcium absorption during coccidial infections in chicks. Poult Sci. (1973) 52:854–7. 10.3382/ps.0520854 4754042

[101] Van Der Klis JD Verstegen MWA De Wit W. Absorption of minerals and retention time of dry matter in the gastrointestinal tract of broilers. Poult Sci. (1990) 69:2185–94. 10.3382/ps.0692185 2084676

[102] Watson BC Matthews JO Southern LL Shelton JL The interactive effects of *Eimeria acervulina* infection and phytase for broiler chicks 1. Poult Sci. (2005) 84:910–3. 10.1093/ps/84.6.910 15971529

[103] Mansoori B Modirsanei M Nodeh H Rahbari S. The interactive effect of phytase and coccidia on the gross lesions as well as the absorption capacity of intestine in broilers fed with diets low in calcium and available phosphorous. Vet Parasitol. (2010) 168:111–5. 10.1016/j.vetpar.2009.10.018 19942351

[104] Fox MC Brown DR Southern LL. Effect of dietary buffer additions on gain, efficiency, duodenal pH, and copper concentration in liver of *Eimeria acervulina*-infected chicks. Poult Sci. (1987) 66:500–4. 10.3382/ps.0660500 3601861

[105] Kiarie E Woyengo T Nyachoti CM. Efficacy of new 6-phytase from *Buttiauxella* spp. on growth performance and nutrient retention in broiler chickens fed corn soybean meal-based diets Asian-Australas. J Anim Sci. (2015) 28:1479–87. 10.5713/ajas.15.0059 26323404 PMC4554856

[106] Shaw AL Van Ginkel FW Macklin KS Blake JP. Effects of phytase supplementation in broiler diets on a natural *Eimeria* challenge in naive and vaccinated birds. Poult Sci. (2011) 90:781–90. 10.3382/ps.2010-01158 21406363

[107] Walk CL Cowieson AJ Remus JC Novak CL Mcelroy AP. Effects of dietary enzymes on performance and intestinal goblet cell number of broilers exposed to a live coccidia oocyst vaccine. Poult Sci. (2011) 90:91–8. 10.3382/ps.2010-00760 21177448

[108] Nyachoti CM Omogbenigun FO Rademacher M Blank G. Performance responses and indicators of gastrointestinal health in early-weaned pigs fed low-protein amino acid-supplemented diets. J Anim Sci. (2006) 84:125–34. 10.2527/2006.841125x 16361499

[109] Ruff MD Johnson JK Dykstra DD Reid WM. Effects of *Eimeria acervulina* on intestinal pH in conventional and gnotobiotic chickens. Avian Dis. (1974) 18:96–104. 10.2307/1589247 4205348

[110] Abbas RZ Munawar SH Manzoor Z Iqbal Z Khan MN Saleemi MK Anticoccidial effects of acetic acid on performance and pathogenic parameters in broiler chickens challenged with *Eimeria tenella*. Pesqui Vet Brasil. (2011) 31:99–103. 10.1590/S0100-736X2011000200001

[111] Bradley GL Savage TF Timm KI. The effects of supplementing diets with *Saccharomyces cerevisiae* var. boulardii on male poult performance and ileal morphology. Poult Sci. 73:1766–70. 10.3382/ps.0731766 7862617

[112] Patterson JA Burkholder KM. Application of prebiotics and probiotics in poultry production. Poult Sci. (2003) 82:627–31. 10.1093/ps/82.4.627 12710484

[113] Liu S-Q Tsao M. Enhancement of survival of probiotic and non-probiotic lactic acid bacteria by yeasts in fermented milk under non-refrigerated conditions. Int J Food Microbiol. (2009) 135:34–8. 10.1016/j.ijfoodmicro.2009.07.017 19666198

[114] Suharja AAS Henriksson A Liu S-Q Impact of *Saccharomyces cerevisiae* on viability of probiotic *Lactobacillus rhamnosus* in fermented milk under ambient conditions. J Food Proc Preserv. (2014) 38:326–337. 10.1111/j.1745-4549.2012.00780.x

[115] Xie N Zhou T Li B Kefir yeasts enhance probiotic potentials of Lactobacillus paracasei H9: the positive effects of coaggregation between the two strains. Food Res Int. (2012) 45:394–401. 10.1016/j.foodres.2011.10.045

[116] Roto SM Rubinelli PM Ricke SC. An introduction to the avian gut microbiota and the effects of yeast-based prebiotic-type compounds as potential feed additives. Front Vet Sci. (2015) 2:28. 10.3389/fvets.2015.00028 26664957 PMC4672232

[117] Foligné B Dewulf J Vandekerckove P Pignède G Pot B. Probiotic yeasts: anti-inflammatory potential of various non-pathogenic strains in experimental colitis in mice. World J Gastroenterol. (2010) 16:2134–45. 10.3748/wjg.v16.i17.2134 20440854 PMC2864839

[118] Van Der Aa Kühle A Skovgaard K Jespersen L. *In vitro* screening of probiotic properties of *Saccharomyces cerevisiae* var. boulardii and food-borne *Saccharomyces cerevisiae* strains. Int J Food Microbiol. (2005) 101:29–39. 10.1016/j.ijfoodmicro.2004.10.039 15878404

[119] Czerucka D Rampal P. Experimental effects of Saccharomyces boulardii on diarrheal pathogens. Microb Infect. (2002) 4:733–9. 10.1016/S1286-4579(02)01592-7 12067833

[120] Czerucka D Piche T Rampal P. Review article: yeast as probiotics–*Saccharomyces boulardii*. Aliment Pharmacol Ther. (2007) 26:767–78. 10.1111/j.1365-2036.2007.03442.x 17767461

[121] Zanello G Meurens F Berri M Salmon H. *Saccharomyces boulardii* effects on gastrointestinal diseases. Curr Issues Mol Biol. (2009) 11:47–8. 10.21775/cimb.011.047 18780946

[122] Geyik MF Aldemir M Hosoglu S Ayaz C Satilmis S Buyukbayram H. The effects of *Saccharomyces boulardii* on bacterial translocation in rats with obstructive jaundice. Ann R Coll Surg Engl. (2006) 88:176–80. 10.1308/003588406X94986 16551414 PMC1964040

[123] Karen M Yuksel O Akyürek N Ofluoglu E Çaglar K Sahin TT. Probiotic agent *Saccharomyces boulardii* reduces the incidence of lung injury in acute necrotizing pancreatitis induced rats. J Surg Res. (2010) 160:139–44. 10.1016/j.jss.2009.02.008 19375719

[124] Line JE Bailey JS Cox NA Stern NJ Tompkins T. Effect of yeast-supplemented feed on *Salmonella* and *Campylobacter* populations in broilers. Poult Sci. (1998) 77:405–10. 10.1093/ps/77.3.405 9521452

[125] Dantán-González E Quiroz-Castañeda RE Cobaxin-Cárdenas M Valle-Hernández J Gama-Martínez Y Tinoco-Valencia JR. Impact of *Meyerozyma guilliermondii* isolated from chickens against *Eimeria* sp. protozoan, an *in vitro* analysis. BMC Vet Res. (2015) 11:278. 10.1186/s12917-015-0589-0 26552648 PMC4640389

[126] Lin G-J Liu T-Y Tseng Y-Y Chen Z-W You C-C Hsuan S-L. Yeast-expressed classical swine fever virus glycoprotein E2 induces a protective immune response. Vet Microbiol. (2009) 139:369–74. 10.1016/j.vetmic.2009.06.027 19625145

[127] Arnold M Durairaj V Mundt E Schulze K Breunig KD Behrens S-E. Protective vaccination against infectious bursal disease virus with whole recombinant *Kluyveromyces lactis* yeast expressing the viral VP2 subunit. PLoS ONE. (2012) 7:e42870. 10.1371/journal.pone.0042870 23024743 PMC3443089

[128] Sathish K Sriraman R Subramanian BM Rao NH Balaji K Narasu ML. Plant expressed EtMIC2 is an effective immunogen in conferring protection against chicken coccidiosis. Vaccine. (2011) 29:9201–8. 10.1016/j.vaccine.2011.09.117 21986219

[129] Sun H Wang L Wang T Zhang J Liu Q Chen P. Display of *Eimeria tenella* EtMic2 protein on the surface of *Saccharomyces cerevisiae* as a potential oral vaccine against chicken coccidiosis. Vaccine. (2014) 32:1869–76. 10.1016/j.vaccine.2014.01.068 24530147

[130] Elaine-Rose M Long PL Immunity to coccidiosis: protective effects of transferred serum and cells investigated in chick embryos infected with *Eimeria tenella*. Parasitology. (2009) 63:299–313. 10.1017/S0031182000079610 5129805

[131] Shivaramaiah C Barta JR Hernandez-Velasco X Téllez G Hargis B Coccidiosis: recent advancements in the immunobiology of Eimeria species, preventive measures, and the importance of vaccination as a control tool against these Apicomplexan parasites. Vet Med. (2014) 5:23–34. 10.2147/VMRR.S57839 PMC7337151 32670843

[132] Wallach M. Role of antibody in immunity and control of chicken coccidiosis. Trends Parasitol. (2010) 26:382–7. 10.1016/j.pt.2010.04.004 20452286

[133] Carver J. Dietary nucleotides: effects on the immune and gastrointestinal systems. Acta Paediatr. (1999) 88:83–8. 10.1111/j.1651-2227.1999.tb01306.x 10569229

[134] Hess JR Greenberg NA. The role of nucleotides in the immune and gastrointestinal systems. Nutr Clin Pract. (2012) 27:281–94. 10.1177/0884533611434933 22392907

[135] Jung B Batal AB. Effect of dietary nucleotide supplementation on performance and development of the gastrointestinal tract of broilers. Br Poult Sci. (2012) 53:98–105. 10.1080/00071668.2012.659654 22404810

[136] Alizadeh M Rodriguez-Lecompte JC Yitbarek A Sharif S Crow G Slominski BA. Effect of yeast-derived products on systemic innate immune response of broiler chickens following a lipopolysaccharide challenge. Poult Sci. (2016) 95:2266–73. 10.3382/ps/pew154 27143776

[137] Navarro J Maldonado J Narbona E Ruiz-Bravo A García Salmerón JL Molina JA. Influence of dietary nucleotides on plasma immunoglobulin levels and lymphocyte subsets of preterm infants. BioFactors. (1999) 10:67–76. 10.1002/biof.5520100108 10475592

[138] Yu VYH. Scientific rationale and benefits of nucleotide supplementation of infant formula. J Paediatr Child Health. (2002) 38:543–49. 10.1046/j.1440-1754.2002.00056.x 12410863

[139] Salaheen S Kim S-W Haley BJ Van Kessel JAS Biswas D. Alternative growth promoters modulate broiler gut microbiome and enhance body weight gain. Front Microbiol. (2017) 8:2088. 10.3389/fmicb.2017.02088 29123512 PMC5662582

[140] Holck P Sletmoen M Stokke BT Permin H Norn S. Potentiation of histamine release by microfungal (1 → 3)- and (1 → 6)-β-D-glucans. Basic Clin Pharmacol Toxicol. (2007) 101:455–8. 10.1111/j.1742-7843.2007.00140.x 17927691

[141] Volman JJ Ramakers JD Plat J. Dietary modulation of immune function by β-glucans. Physiol Behav. (2008) 94:276–84. 10.1016/j.physbeh.2007.11.045 18222501

[142] Elmer GW Mcfarland LV. Suppression by *Saccharomyces boulardii* of toxigenic *Clostridium difficile* overgrowth after vancomycin treatment in hamsters. Antimicrob Agents Chemother. (1987) 31:129. 10.1128/AAC.31.1.129 3566236 PMC174670

[143] Ghoneum M Wang L Agrawal S Gollapudi S. Yeast therapy for the treatment of breast cancer: a nude mice model study. In Vivo. (2007) 21:251–8. Available online at: http://iv.iiarjournals.org/content/21/2/251.long 17436573

[144] Xue G-D Wu S-B Choct M Swick RA. Effects of yeast cell wall on growth performance, immune responses and intestinal short chain fatty acid concentrations of broilers in an experimental necrotic enteritis model. Anim Nutr. (2017) 3:399–405. 10.1016/j.aninu.2017.08.002 29767160 PMC5941278

[145] Hooge DM Meta-analysis of broiler chicken pen trials evaluating dietary mannan oligosaccharide, 1993-2003. Int J Poult Sci. (2004) 3:163–74. 10.3923/ijps.2004.163.174

[146] Shashidhara R Devegowda G. Effect of dietary mannan oligosaccharide on broiler breeder production traits and immunity. Poult Sci. (2003) 82:1319–25. 10.1093/ps/82.8.1319 12943304

[147] M'sadeq SA Wu S-B Choct M Forder R Swick RA. Use of yeast cell wall extract as a tool to reduce the impact of necrotic enteritis in broilers. Poult Sci. (2015) 94:898–905. 10.3382/ps/pev035 25762162

[148] Rose ME. Immune responses in infections with coccidia: macrophage activity. Infect Immun. (1974) 10:862–71. 4426710 10.1128/iai.10.4.862-871.1974 PMC423033

[149] Lu Z Thanabalan A Leung H Akbari Moghaddam Kakhki R Patterson R Kiarie EG. The effects of feeding yeast bioactives to broiler breeders and/or their offspring on growth performance, gut development, and immune function in broiler chickens challenged with *Eimeria*. Poult Sci. (2019) 98:6411–21. 10.3382/ps/pez479 31504867 PMC6870552

[150] Girard F Fort G Yvoré P Quéré P Kinetics of specific immunoglobulin A, M and G production in the duodenal and caecal mucosa of chickens infected with *Eimeria acervulina* or *Eimeria tenella*. Int J Parasitol. (1997) 27:803–9. 10.1016/S0020-7519(97)00044-1 9279583

[151] Yun CH Lillehoj HS Lillehoj EP. Intestinal immune responses to coccidiosis. Dev Comp Immunol. (2000) 24:303–24. 10.1016/S0145-305X(99)00080-4 10717295

[152] Gómez-Verduzco G Cortes-Cuevas A López-Coello C Avila-González E Nava GM. Dietary supplementation of mannan-oligosaccharide enhances neonatal immune responses in chickens during natural exposure to *Eimeria* spp. Acta Vet Scand. (2009) 51:11. 10.1186/1751-0147-51-11 19298670 PMC2667520

[153] Elmusharaf MA Bautista V Nollet L Beynen AC Effect of a mannanoligosaccharide preparation on *Eimeria tenella* infection in broiler chickens. Int J Poult Sci. (2006) 5:583–8. 10.3923/ijps.2006.583.588

[154] Elmusharaf MA Peek HW Nollet L Beynen AC The effect of an in-feed mannanoligosaccharide preparation (MOS) on a coccidiosis infection in broilers. Anim Feed Sci Technol. (2007) 134:347–54. 10.1016/j.anifeedsci.2006.11.022

[155] Shanmugasundaram R Sifri M Selvaraj RK. Effect of yeast cell product (CitriStim) supplementation on broiler performance and intestinal immune cell parameters during an experimental coccidial infection1. Poult Sci. (2013) 92:358–63. 10.3382/ps.2012-02776 23300301

[156] Lu HA Adedokun S Adeola LM Ajuwon K Anti-inflammatory effects of non-antibiotic alternatives in coccidia challenged broiler chickens. J Poult Sci. (2014) 51:14–21. 10.2141/jpsa.0120176

[157] Calder PC Krauss-Etschmann S De Jong EC Dupont C Frick JS Frokiaer H. Early nutrition and immunity - progress and perspectives. Br J Nutr. (2006) 96:774–90. 10.1079/BJN20061917 17010239

[158] Reynolds LP Caton JS. Role of the pre- and post-natal environment in developmental programming of health and productivity. Mol Cell Endocrinol. (2012) 354:54–9. 10.1016/j.mce.2011.11.013 22154989 PMC3306485

[159] Rudrappa SG Humphrey BD. Energy metabolism in developing chicken lymphocytes is altered during the embryonic to posthatch transition. J Nutr. (2007) 137:427–32. 10.1093/jn/137.2.427 17237322

[160] Smith NC Wallach M Miller CM Braun R Eckert J. Maternal transmission of immunity to Eimeria maxima: western blot analysis of protective antibodies induced by infection. Infect Immun. (1994) 62:4811–7. 7927759 10.1128/iai.62.11.4811-4817.1994 PMC303191

[161] Rose ME. Immunity to coccidiosis: maternal transfer in Eimeria maxima infections. Parasitology. (2009) 65:273–82. 10.1017/S0031182000045054 4680537

